# Neuropsychiatric Safety of GLP-1 Receptor Agonists and Tirzepatide in Adults with Type 2 Diabetes Mellitus or Obesity

**DOI:** 10.5041/RMMJ.10585

**Published:** 2026-10-26

**Authors:** Het Tushar Jani, Helisse Levine, Abhiraj Patel, Richa Trivedi, Xitij Dineshkumar Patel

**Affiliations:** 1 Master of Public Health Program, Long Island University, Brooklyn, New York, USA; 2 Frank H. Netter MD School of Medicine, Quinnipiac University, North Haven, Connecticut, USA; 3 St. Vincent’s Medical Center, Hartford HealthCare, Bridgeport, Connecticut, USA

**Keywords:** Depression, glucagon-like peptide-1 receptor agonists, obesity, suicidal ideation, tirzepatide, type 2 diabetes mellitus

## Abstract

**Objective:**

Neuropsychiatric safety concerns about glucagon-like peptide-1 receptor agonists (GLP-1 RAs) and tirzepatide prompted regulatory review by the European Medicines Agency (EMA) and the US Food and Drug Administration (FDA). We synthesized the contemporary evidence in adults with type 2 diabetes mellitus or obesity, emphasizing study design, risk of bias, and the distinction between signal detection and causal inference.

**Methods:**

The PubMed/MEDLINE, Embase, Web of Science, and Scopus databases were searched without a lower date limit through March 15, 2026 (English, adults ≥18 years). The review was reported with reference to the Preferred Reporting Items for Systematic Reviews and Meta-Analyses (PRISMA) 2020, and the PRISMA-S and PRISMA-Harms extensions. Evidence was grouped into prespecified categories: randomized syntheses, comparative observational studies and target-trial emulations, outlier non-active-comparator studies, pharmacovigilance analyses, and regulatory reviews. Risk of bias was appraised with the Cochrane Risk of Bias 2 tool and with Risk of Bias in Non-randomised Studies of Interventions; certainty was summarized using Grading of Recommendations Assessment, Development and Evaluation.

**Results:**

Twenty studies and two regulatory reviews met the inclusion criteria. Randomized evidence, including two large 2025 meta-analyses and post hoc pooled analyses of semaglutide and tirzepatide, showed no increased risk of suicidality, depression, or anxiety, though psychiatric exclusion criteria limited generalizability. Active-comparator cohorts from the United States, Scandinavia, and the United Kingdom likewise showed no clear increase after confounding control. Passive-comparator and disproportionality analyses generated signals but could not separate drug effects from baseline psychiatric risk, obesity-related distress, or reporting bias. The EMA and FDA conclusions do not support a causal class-wide association between GLP-1 RAs and suicidal ideation or behavior.

**Conclusions:**

Evidence does not support a clinically meaningful increase in neuropsychiatric harm with GLP-1 RAs or tirzepatide in the populations studied. Surveillance remains warranted for patients with substantial psychiatric morbidity, for obesity-only populations, and for long-term rare outcomes.

## INTRODUCTION

Glucagon-like peptide-1 receptor agonists (GLP-1 RAs) and tirzepatide, a dual glucose-dependent insulinotropic polypeptide/GLP-1 RA, are now widely used to treat type 2 diabetes mellitus (T2DM) and obesity because of their glycemic, weight, and cardiometabolic benefits.^1^ Regulatory concern about suicidality emerged in 2023 after spontaneous reports were published involving semaglutide- and liraglutide-containing products,^2^,^3^ prompting parallel reviews by the European Medicines Agency (EMA)^4^ and the US Food and Drug Administration (FDA).^5^

The central nervous system distribution of GLP-1 receptors and the known interactions of incretin signaling with stress, reward, eating behavior, inflammation, and cognition make neuropsychiatric safety concerns biologically plausible enough to warrant formal review,^1^,^6^ but these mechanistic considerations do not establish causality. Mechanistic studies have additionally implicated GLP-1 signaling in mood-relevant pathways, including possible antidepressant-like and neuroprotective effects mediated through synaptic and neuroinflammatory mechanisms. 7,8

The literature is now large enough that study design matters more than headline direction. Rare-event randomized controlled trial (RCT) syntheses, active-comparator cohort studies, passive-comparator electronic health record studies, and pharmacovigilance analyses answer different questions and carry different bias structures. The present review reorganizes findings by evidence type and prioritizes original trial-based meta-analyses, comparative observational studies, and official regulatory appraisals.

## METHODS

### Protocol and Reporting

This review was reported with reference to the Preferred Reporting Items for Systematic Reviews and Meta-Analyses (PRISMA) 2020 statement and the PRISMA-S (search strategy) and PRISMA-Harms extensions.^9^–^11^ A review protocol was drafted before screening began but was not prospectively registered in PROSPERO. The review question was refined as the EMA and FDA regulatory reviews were published during the search period; consequently, registration was not completed before screening began. The protocol, eligibility criteria, and search strategy were finalized before full-text screening. The protocol, search strategy, and screening logs are available from the corresponding author on reasonable request.

### Search Strategy

The PubMed/MEDLINE, Embase, Web of Science, and Scopus databases were systematically searched from inception (i.e. without a lower date limit) through March 15, 2026. Search strategies combined controlled vocabulary (e.g. Medical Subject Headings) and free-text terms for GLP-1 RAs and tirzepatide (exenatide, liraglutide, lixisenatide, dulaglutide, semaglutide, albiglutide, tirzepatide) with neuropsychiatric outcomes (depression, anxiety, suicide, suicidal ideation, self-harm, mood disorders, eating disorders, psychiatric adverse events). The following limits were applied: English language, human subjects, and adults (≥18 years). No restrictions were placed on study design. Reference lists of included studies and relevant systematic reviews were also screened; no additional eligible records were identified. The complete database-specific search strings are provided in Supplement A.

### Eligibility Criteria

Studies were included if they met all of the following criteria:

- Adult (≥18 years) human populations with T2DM, overweight, or obesity;
- Exposure to a GLP-1 RA (exenatide, liraglutide, lixisenatide, dulaglutide, semaglutide, albiglutide) or tirzepatide;
- Reported neuropsychiatric outcomes including death by suicide, suicidal ideation, suicide attempts, self-harm, depression, anxiety, mood disorders, eating disorders, or other psychiatric adverse events;
- Study designs including meta-analyses and pooled analyses of RCTs, cohort studies, case-control studies, target-trial emulations, case-time-control studies, post-marketing surveillance, pharmacovigilance analyses, and official regulatory reviews; individual RCTs not already synthesized in a meta-analysis or pooled analysis were not separately included, as the relevant trial-level evidence has already been incorporated into recent meta-analyses;
- Published in English in a peer-reviewed journal or as a formal regulatory communication.

Studies were excluded if they were conference abstracts, editorials or opinion pieces, animal-only studies, narrative reviews without primary data extraction, pediatric or adolescent-only populations, or did not report relevant neuropsychiatric outcomes.

### Study Selection and Data Extraction

All records identified through database searches were exported to Rayyan, a systematic review screening platform,^12^ for duplicate removal and screening. Titles and abstracts were independently screened by two reviewers (R.T. and X.D.P.) against predefined eligibility criteria, followed by independent full-text review by the same two reviewers. Discrepancies at either stage were resolved by discussion or, when not resolved by discussion, by consultation with a third reviewer (H.T.J.). Reasons for full-text exclusion were documented. Data were extracted in duplicate by R.T. and X.D.P. using a standardized form covering study design, country, population characteristics, exposure, comparator, neuropsychiatric outcomes, effect estimates, follow-up, and risk-of-bias considerations, with verification by H.T.J. The selection process is presented in Figure 1.

**Figure 1:**
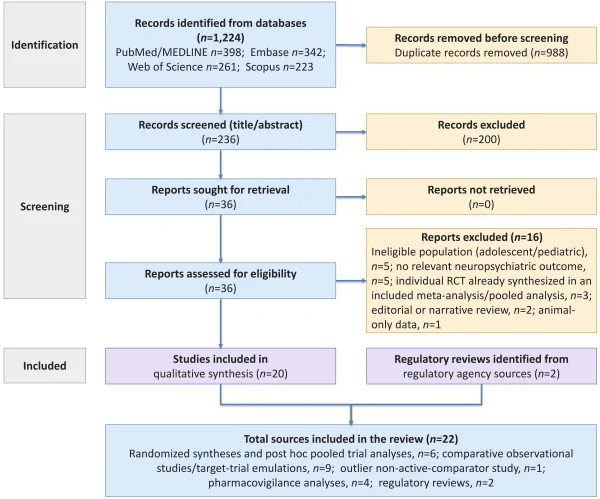
PRISMA 2020 Flow Diagram of the Identification, Screening, Eligibility Assessment, and Inclusion of Evidence Sources on the Neuropsychiatric Safety of GLP-1 Receptor Agonists and Tirzepatide.

### Risk of Bias and Certainty Assessment

Randomized controlled trials and post hoc trial analyses were appraised with the Cochrane Risk of Bias 2 tool;^13^ comparative observational studies, including target-trial emulations and case-time-control designs, were appraised with Risk of Bias in Non-randomised Studies of Interventions.^14^ Pharmacovigilance and disproportionality analyses were not formally scored using standardized tools because of the inherent limitations of spontaneous reporting systems (no denominator data, reporting bias, notoriety effects, confounding by indication, and inability to estimate incidence); these studies were qualitatively appraised and treated as signal-generating rather than causal. Trial-based meta-analyses were not re-appraised with the Cochrane Risk of Bias 2 tool, because they are syntheses of individual trials rather than primary randomized controlled trials; for these, risk of bias as judged within each source synthesis was considered alongside the certainty assessment. Per-study risk-of-bias judgments are reported in Supplement B. Certainty of evidence for the principal conclusions was summarized using Grading of Recommendations Assessment, Development and Evaluation (GRADE).^15^

### Synthesis

Evidence was grouped into five prespecified categories: (1) meta-analyses and pooled analyses of RCTs; (2) comparative observational studies and target-trial emulations, comprising active-comparator cohorts, two broad non-active-comparator cohorts, and one self-controlled case-time-control analysis; (3) outlier non-active-comparator observational studies; (4) pharmacovigilance/disproportionality analyses; and (5) regulatory reviews. Because harm ascertainment, comparator structure, follow-up, and confounding control differed fundamentally across designs, no single pooled estimate was produced spanning RCTs, observational cohorts, and pharmacovigilance studies; this choice follows current methodological guidance for harms reviews.^11^

## RESULTS

### Study Selection

A total of 1,224 records were identified through database searches. Following duplicate removal and screening, 20 studies met the inclusion criteria. In addition, 2 formal regulatory reviews were identified from regulatory agency sources, giving 22 evidence sources in total (Figure 1).^4^,^5^ The characteristics and main findings of the included studies and regulatory reviews are summarized in Table 1.

**Table 1:** Characteristics and Main Findings of Included Studies and Regulatory Reviews.

Evidence Type	Study/ Source	Population/Sample or Scope	Comparator	Main Outcome(s)	Key Estimate(s)
RCT pooled analysis	O’Neil et al. (2017)^16^	5,325 adults with overweight or obesity, pooled across the phase 2/3a SCALE trials	Placebo	Depression, anxiety, suicidality (CSSRS, PHQ-9)	Depression 2.1 vs 2.1/100 PY; SI 1.0% vs 1.0%
RCT meta-analysis	Silverii et al. (2024)^17^	31 RCTs; 84,713 participants (22 trials in T2DM, 9 in obesity)	Placebo / active comparators	Any psychiatric disorder; SB	OR 0.97; OR 0.86
RCT meta-analysis	Pierret et al. (2025)^18^	80 RCTs; 107,860 participants with overweight or obesity and/or T2DM	Placebo	Psychiatric AEs; depressive symptoms; QoL	No increase; QoL favored GLP-1 RAs
RCT meta-analysis	Ebrahimi et al. (2025)^19^	27 placebo-controlled RCTs; 59,396 participants (32,354 GLP-1 RA, 27,042 placebo)	Placebo	Suicide / self-harm	RR 0.76 (0.48–1.21); 0.047 vs 0.042/100 PY
RCT post hoc pooled	Wadden et al. (2024)^20^	3,377 participants pooled from STEP 1, 2, and 3, and 304 participants from STEP 5 (STEP 2 participants also had T2DM)	Placebo	PHQ-9; C-SSRS; AEs	PHQ-9 −0.56 at week 68; no SI/SB excess
RCT post hoc pooled	Wadden et al. (2026)^21^	4,056 participants with obesity or overweight (SURMOUNT-1, -2 and -3)	Placebo	PHQ-9; C-SSRS; AEs	SI 0.6% vs 0.6%; PHQ-9 −0.6
Target-trial emulation	Tang et al. (2024)^22^	GLP-1 RA initiators: 21,807 matched with SGLT2i initiators; 21,402 matched with DPP-4i initiators	Active	SI/SB	HR 1.07 vs SGLT2i; HR 0.94 vs DPP-4i
Active-comparator cohort	Ueda et al. (2024)^23^	124,517 GLP-1 RA initiators, compared with SGLT2i initiators	SGLT2i	Suicide death; composite	HR 1.25 (0.83–1.88); composite HR 0.83
Active-comparator cohort	Shapiro et al. (2025)^24^	36,082 GLP-1 RA versus 234,028 DPP-4i initiators; 32,336 GLP-1 RA versus 96,212 SGLT2i initiators	DPP-4i / SGLT2i	Suicidality composite	HR 1.02 vs DPP-4i; 0.91 vs SGLT2i
Target-trial emulation	Tang et al. (2025)^25^	GLP-1 RA initiators: 14,665 matched with SGLT2i initiators; 13,711 matched with DPP-4i initiators	Active	Incident depression	HR 1.07 vs SGLT2i; HR 0.90 vs DPP-4i
Propensity-matched cohort	De Giorgi et al. (2024)^26^	Propensity-matched pairs of semaglutide versus comparator: 23,386 versus sitagliptin; 22,584 versus empagliflozin; 19,206 versus glipizide	Active	22 neuropsychiatric outcomes	No psychiatric harm; favorable cognitive/nicotine signals
Broad EHR cohort	Wang et al. (2024)^27^	240,618 patients with obesity; replication cohort of 1,589,855 patients with T2DM	Non-GLP-1 anti-obesity / antidiabetic	Incident/recurrent SI	Incident HR 0.27; recurrent HR 0.44 (obesity)
Earlier cohort	Gamble et al. (2018)^28^	501 GLP-1 RA users in the UK CPRD, compared with users of other glucose-lowering drugs	Other glucose-lowering	Depression/self-harm	No clear excess risk
Case-time-control	Bezin et al. (2025)^29^	1,102 cases of suicide or suicide attempt (French SNDS)	Self-controlled with time controls	Suicide/suicide attempt	Adjusted case-time-control ratio 0.62 (0.51–0.75)
Tirzepatide real-world cohort	Yu et al. (2025)^30^	16,321 propensity-matched pairs, tirzepatide versus non-GLP-1 anti-obesity drugs (US TriNetX; 29% with T2DM)	Non-GLP-1 anti-obesity drugs	SI/attempts	aHR 0.52 (0.28–0.91); 17 vs 33 events
Outlier observation al cohort	Kornelius et al. (2024)^31^	162,253 patients with obesity treated with liraglutide or semaglutide, compared with non-users	Non-users	Depression, anxiety, SB	Markedly elevated risks reported
EudraVigilance analysis	Tobaiqy and Elkout (2024)^32^	31,444 individual case safety reports, of which 372 were psychiatric	None	Psychiatric AEs	Depression 50%; anxiety 39%; SI 19.6% of psychiatric reports
FAERS analysis	Chen et al. (2024)^33^	181,238 GLP-1 RA reports, of which 8,240 were psychiatric	None	Psychiatric AEs	Multiple signals; median onset 31 days
WHO disproportionality	Schoretsanitis et al. (2024)^34^	Reports for semaglutide and liraglutide in the WHO global pharmacovigilance database	All drugs	SI; self-injurious ADRs	Semaglutide SI ROR 1.45; +antidepressant ROR 4.45; +benzodiazepine ROR 4.07
FAERS neuropsychiatric analysis	Lu et al. (2025)^35^	25,110 GLP-1 RA-related neuropsychiatric adverse-event reports	None	Neuropsychiatric AEs	Semaglutide weight-loss suicide-related ROR 2.55
Regulatory review	European Medicines Agency (2024)^4^	Class-wide review of GLP-1 RAs	Regulatory synthesis	Suicidal/self-injurious thoughts/actions	No causal association supported
Regulatory review	US Food and Drug Administration (2026)^5^	91 placebo-controlled trials; *n*=107,910 plus Sentinel cohort, *n*=2,243,138	Placebo and active comparator	SI/behavior; psychiatric AEs	No increased risk; warning removal requested

ADR(s), adverse drug reaction(s); AE(s), adverse event(s); aHR, adjusted hazard ratio; C-SSRS, Columbia Suicide Severity Rating Scale; DPP-4i, dipeptidyl peptidase-4 inhibitor; EHR, electronic health record; EudraVigilance, European Union adverse event reporting database; FAERS, FDA Adverse Event Reporting System; GLP-1 RAs, glucagon-like peptide-1 receptor agonists; HR, hazard ratio; OR, odds ratio; PHQ-9, Patient Health Questionnaire-9; PY, person-years; QoL, quality of life; RCT(s), randomized controlled trial(s); ROR, reporting odds ratio; RR, risk ratio; SB, suicidal behavior; SCALE, liraglutide weight-management trial program; SGLT2i, sodium-glucose co-transporter 2 inhibitor; SI, suicidal ideation; SNDS, Système National des Données de Santé (French national health data system); T2DM, type 2 diabetes mellitus; UK CPRD, United Kingdom Clinical Practice Research Datalink; WHO, World Health Organization.

### Meta-analyses and Pooled Analyses of Randomized Controlled Trials

The randomized evidence base was consistently reassuring. O’Neil and colleagues pooled phase 2 and 3a liraglutide weight-management trials from the multinational SCALE trial program.^16^ Participants were adults with obesity or overweight (body mass index ≥27 kg/m^2^ with weight-related comorbidities). Depression and anxiety event rates were low and similar between groups. A small numerical imbalance in spontaneously reported suicidal ideation or behavior did not persist in prospective assessments using the Columbia Suicide Severity Rating Scale (C-SSRS) and Patient Health Questionnaire-9 (PHQ-9).^16^

In an RCT meta-analysis by Silverii and colleagues, 31 trials involving 84,713 participants were included: 22 trials involved participants with T2DM, and 9 involved participants with obesity. Glucagon-like peptide-1 receptor agonists were not associated with an increased risk of psychiatric disorders (odds ratio [OR] 0.97), suicidal behavior (OR 0.86), depression, or anxiety compared with placebo or active comparators.^17^ In a larger placebo-controlled synthesis by Pierret and colleagues, 80 RCTs involving 107,860 participants were included. The trials enrolled adults with overweight or obesity and/or T2DM across sites in North America, Europe, and Asia. No increase in serious or non-serious psychiatric adverse events was observed, while mental and physical health-related quality of life and eating-related outcomes modestly favored GLP-1 RA therapy.^18^ Ebrahimi and colleagues conducted a meta-analysis of 27 placebo-controlled RCTs of at least 6 months’ duration in adults with diabetes or obesity. The analysis included 32,354 GLP-1 RA-treated and 27,042 placebo-treated participants from multinational trial sites. Suicide and self-harm events were very rare, with weighted incidence rates of 0.047 and 0.042 per 100 person-years in the GLP-1 RA and placebo groups, respectively. There was no significant difference between groups (risk ratio 0.76; 95% CI 0.48–1.21).^19^ The three syntheses differed mainly in scope and eligibility criteria rather than in overall conclusions. Silverii and colleagues restricted their analysis to trials with dedicated psychiatric-outcome reporting.^17^ Pierret and colleagues included the broadest range of psychiatric, cognitive, and quality-of-life outcomes, regardless of trial duration,^18^ whereas Ebrahimi and colleagues focused specifically on suicide and self-harm in trials of at least 6 months.^19^ The different numbers of trials and participants therefore reflect differing eligibility criteria applied to a substantially overlapping body of published randomized controlled trials.

Drug-specific trial analyses were consistent with the broader randomized evidence. In the analyses of STEP 1, 2, 3, and 5 trials, Wadden and colleagues evaluated semaglutide 2.4 mg in adults with overweight or obesity at multicenter international trial sites.^20^ Participants in STEP 2 also had T2DM. Semaglutide was not associated with increased suicidal ideation or behavior compared with placebo. The estimated treatment difference in PHQ-9 score modestly favored semaglutide (−0.56), and shifts toward more severe depressive symptom categories were less common with active treatment.^20^ In post hoc analyses of SURMOUNT-1, -2, and -3, tirzepatide was evaluated in adults with overweight or obesity, with and without T2DM, at multinational trial sites; SURMOUNT-2 enrolled participants with T2DM. Tirzepatide was not associated with a higher risk of depression or suicidality in adults without major baseline psychopathology. Suicidal ideation occurred in 0.6% of both tirzepatide-treated and placebo-treated participants, and depressive symptom scores again modestly favored active treatment (PHQ-9 difference −0.6).^21^ The principal limitation across this RCT evidence is limited external validity. Major psychiatric illness, recent suicidality, and severe depressive syndromes were often exclusion criteria, limiting the applicability of these findings to patients with these conditions.

### Comparative Observational Studies and Target-trial Emulations

Real-world evidence using an active comparator—that is, drug-versus-drug comparisons designed to reduce confounding by indication—also generally showed no clear increase in risk after adjustment for confounding. In a US Medicare target-trial emulation of older adults with T2DM, Tang and colleagues reported no increased risk of suicidal ideation or behaviors for GLP-1 RA users compared with sodium-glucose co-transporter 2 inhibitor (SGLT2i) or dipeptidyl peptidase-4 inhibitor (DPP-4i) users. The corresponding hazard ratios were 1.07 and 0.94, respectively, with small absolute rate differences.^22^

In the Scandinavian register-based active-comparator new-user study by Ueda and colleagues, conducted in Sweden and Denmark among adults with T2DM, GLP-1 RA use was not associated with higher suicide death risk than SGLT2i use. Weighted incidence rates were 0.23 versus 0.18 per 1,000 person-years, and the hazard ratio was 1.25 (95% CI 0.83–1.88).^23^ In the United Kingdom Clinical Practice Research Datalink (CPRD) study by Shapiro and colleagues, conducted in adults with T2DM, crude risk estimates were higher than those for DPP-4i or SGLT2i comparators but attenuated to the null after adjustment for confounding (adjusted hazard ratio [HR] 1.02 versus DPP-4i; 0.91 versus SGLT2i). These findings highlight the importance of comparator choice and baseline psychiatric burden.^24^

Findings for depression varied depending on which comparator drug was used, illustrating that comparator choice can affect the observed direction of effect even within the same source population. In older US Medicare beneficiaries with T2DM, Tang and colleagues found no significant increase in incident depression versus SGLT2i (HR 1.07), while a modestly lower risk was observed versus DPP-4i (HR 0.90).^25^ This pattern does not support a class-wide depressogenic effect. De Giorgi and colleagues used the US TriNetX electronic health record network to compare propensity-matched adults with T2DM receiving semaglutide with those receiving sitagliptin, empagliflozin, and glipizide. They likewise found no increased 12-month risk of adverse psychiatric outcomes. They also reported lower risks for selected cognitive outcomes and nicotine- or tobacco-use disorders, although these potentially beneficial associations remain hypothesis-generating.^26^

Additional observational studies broadly fit that pattern. Wang and colleagues, using the US TriNetX network, found lower observed suicidal ideation risk with semaglutide than with non-GLP-1 comparators in obesity and diabetes cohorts, but the broad non-active-comparator structure makes these estimates more susceptible to residual confounding.^27^ Gamble and colleagues used the CPRD to study 501 patients with T2DM who initiated GLP-1 RA treatment, compared with patients receiving other glucose-lowering therapies. They found no increased risk of depression or self-harm with incretin-based therapies in routine UK practice (HR 1.25, 95% CI 0.63–2.50), although the modest exposed-group sample size limited statistical precision.^28^

A French nationwide case-time-control analysis by Bezin and colleagues used the French national health data system (SNDS) to examine adult GLP-1 RA users, of whom 51% had obesity and 68% had a recent psychiatric history. The analysis showed no evidence of increased risk, with an adjusted case-time-control ratio below 1 overall. In this self-controlled, within-person design, each patient’s exposed and unexposed periods were compared, reducing confounding by fixed characteristics such as baseline psychiatric vulnerability or socioeconomic status. However, the design remains susceptible to time-varying confounding and reverse causation around treatment initiation and has limited ability to estimate absolute risk. These limitations are less pronounced, although not absent, in parallel-group active-comparator cohort studies.^29^ Tirzepatide-specific real-world evidence from Yu and colleagues, using the US TriNetX network, included 226,060 patients with overweight or obesity, 29% of whom had T2DM. Analysis of 16,321 propensity-matched pairs showed no increased suicide-related risk with tirzepatide; instead, the investigators observed a lower adjusted HR versus non-GLP-1 anti-obesity medications (adjusted HR 0.52; 95% CI 0.28–0.91).^30^

### Outlier Observational Evidence

One observational study stood apart from the broader literature. Kornelius and colleagues, drawing on the US TriNetX network, reported sharply elevated risks of psychiatric disorders, depression, anxiety, and suicidal behavior among patients with obesity treated with liraglutide or semaglutide compared with non-users.^31^ Those effect sizes were materially larger than randomized evidence, active-comparator studies, and the eventual regulatory conclusions. Part of this signal is likely attributable to the elevated baseline psychiatric burden of the population studied rather than to a drug effect: adults with obesity and T2DM have poorer psychiatric outcomes at baseline than those without these conditions,^18^ and a passive, non-active-comparator design cannot separate that elevated baseline risk from any effect of treatment itself. Methodologically, this study is best viewed as an outlier because of its passive-comparator structure, vulnerability to residual confounding by indication, uncertainty about psychiatric outcome ascertainment, and the possibility that obesity severity, treatment selection, and healthcare-contact intensity influenced both exposure and event detection. Accordingly, this study was not given decisive weight in the synthesis of the evidence.

### Pharmacovigilance Evidence

Signal-detection studies yielded mixed results and require careful framing. The following three pharmacovigilance databases differ in geographic scope: the European Union adverse event reporting database, the FDA Adverse Event Reporting System (FAERS), and the World Health Organization global pharmacovigilance database; the signals detected in these databases reflect different reporting populations and practices. Tobaiqy and Elkout found 372 psychiatric adverse-event reports among 31,444 European Union adverse event reporting database reports for semaglutide, liraglutide, and tirzepatide, with depression, anxiety, and suicidal ideation among the most commonly reported psychiatric events.^32^ Chen and colleagues found 8,240 psychiatric adverse-event reports among 181,238 FAERS reports related to GLP-1 RAs and detected multiple psychiatric signals, with a median onset of 31 days.^33^ Schoretsanitis and colleagues, using the World Health Organization global pharmacovigilance database, detected a semaglutide-associated disproportionality signal for suicidal ideation (reporting OR [ROR] 1.45) that strengthened in subgroups with co-reported antidepressants (ROR 4.45) or benzodiazepines (ROR 4.07).^34^ Lu and colleagues similarly identified broader neuropsychiatric signals in FAERS, including a suicide-related signal in semaglutide reports from weight-loss populations (ROR 2.55).^35^ Across all spontaneous-reporting analyses, the absence of denominator data precludes incidence estimation and prevents causal attribution; these signals are therefore hypothesis-generating only.

### Regulatory Evidence

Current regulatory assessments do not support a causal class-wide association. In April 2024, the EMA Pharmacovigilance Risk Assessment Committee concluded that available evidence did not support a causal association between GLP-1 RAs and suicidal or self-injurious thoughts and actions.^4^ In January 2026, the FDA went further, requesting removal of suicidal ideation/behavior warning language from obesity-treatment GLP-1 RA labels after a class-wide review that included 91 placebo-controlled trials with 107,910 patients and a Sentinel cohort of 2,243,138 GLP-1 RA and SGLT2i users, neither of which showed increased risk.^5^ An expert commentary in the *BMJ* contextualized this evidence shift, emphasizing that high-quality observational and randomized evidence converge on reassurance even as continued surveillance remains appropriate.^36^ Risk-of-bias and certainty appraisals by evidence group are summarized in Table 2. The GRADE certainty ratings for the principal outcomes are summarized in Table 3.

**Table 2:** Risk-of-Bias, Certainty, and Decision-Relevance Appraisal by Evidence Group.

Evidence Group	Dominant Bias Concerns	Risk-of-bias Judgment	Certainty of Evidence (GRADE)*
RCT post hoc / pooled analyses	Psychiatric exclusion criteria; post hoc harms; rare-event underpowering	Some concerns (RoB 2)	Moderate
RCT meta-analyses	Rare-event ascertainment; heterogeneous harms definitions	Not re-appraised with RoB 2; risk of bias as judged within each source synthesis	Moderate
Active-comparator cohort / target-trial emulations	Residual confounding; outcome misclassification; treatment channeling	Moderate (ROBINS-I)	Moderate
Broad EHR / passive-comparator studies	Comparator imbalance; surveillance bias; obesity-severity confounding	Serious to critical (ROBINS-I)	Low
Pharmacovigilance / disproportionality	Notoriety bias; no denominator data; cannot estimate incidence	Not formally scored (qualitative appraisal only)	Very low
Regulatory reviews	Dependence on quality of underlying data; integration of multiple sources	Not applicable (regulatory evidence synthesis)	Not applicable †

* Certainty ratings in this column are GRADE assessments made at the level of each evidence group; outcome-specific GRADE ratings are given in Table 3.

† Regulatory reviews are not assigned a GRADE certainty rating; they are instead of high decision relevance, because they integrate multiple evidence streams (randomized controlled trials, large electronic health record/Sentinel cohorts, and spontaneous reports) under a formal regulatory risk–benefit framework and directly inform prescribing and labeling decisions. Per-study domain-level RoB 2/ROBINS-I judgments are reported in Supplement B.

EHR, electronic health record; GRADE, Grading of Recommendations Assessment, Development and Evaluation; RoB 2, Cochrane Risk of Bias 2; ROBINS-I, Risk of Bias in Non-randomised Studies of Interventions.

**Table 3:** GRADE Summary of Findings.

Outcome	Effect/Direction	Evidence stream	Certainty (GRADE)	Reason for Downgrading
Suicide death and self-harm (adults with T2DM / obesity)	No class-wide increase; rare events; near-null pooled estimates	RCT meta-analyses; active-comparator cohorts; regulatory reviews	Moderate	Imprecision (rare events)
Incident depression	No class-wide increase; some comparator-sensitive variation	RCT meta-analyses; active-comparator cohorts	Low	Indirectness (heterogeneous outcome definitions); imprecision
Anxiety symptoms / disorder	No class-wide increase	RCT meta-analyses; active-comparator cohorts	Low	Indirectness (heterogeneous ascertainment); imprecision (few dedicated analyses)
Tirzepatide-specific psychiatric outcomes	No signal of increased risk	Post hoc pooled RCT (SURMOUNT); one real-world cohort	Low	Imprecision (few studies); indirectness (limited follow-up)
Pharmacovigilance disproportionality signals	Mixed signals; stronger signals in subgroups with co-reported antidepressants or benzodiazepines	EudraVigilance; FAERS; WHO VigiBase	Very low	No denominator data; reporting and notoriety bias; cannot estimate incidence

**GRADE certainty ratings**: high, moderate, low, very low. **Downgrading domains:** risk of bias, inconsistency, indirectness, imprecision, publication bias. Regulatory reviews are not given a separate row in this table because they are not assigned GRADE certainty ratings; their conclusions integrate multiple evidence streams under a formal risk–benefit framework and are summarized in Table 2 and in the Regulatory Evidence section.

EudraVigilance, European Union adverse event reporting database; FAERS, FDA Adverse Event Reporting System; GRADE, Grading of Recommendations Assessment, Development and Evaluation; RCT, randomized controlled trial; T2DM, type 2 diabetes mellitus; VigiBase, World Health Organization global pharmacovigilance database; WHO, World Health Organization.

## DISCUSSION

### Main Findings

The central conclusion of this review is that the apparent conflict in the literature is largely methodological rather than biological. Randomized evidence and active-comparator cohort studies are directionally concordant and mostly null;^18^,^19^,^22^–^25^ passive-comparator or broad electronic health record studies and pharmacovigilance analyses generate more concerning signals.^31^,^34^,^35^ However, those designs are also more vulnerable to confounding by indication (patients started on treatment because they already carry higher baseline metabolic or psychiatric risk), obesity-related psychosocial distress (weight stigma, body image concerns, and disordered eating that can pre-date and accompany treatment), differential healthcare contact (more frequent clinical encounters among treated patients, increasing the opportunity for a psychiatric diagnosis or adverse event to be captured), stimulated reporting (heightened clinician and patient awareness following the 2023 regulatory alerts, which can inflate spontaneous-report counts independent of any change in true incidence), and comparator mismatch (comparator drugs or non-user groups with a different underlying psychiatric risk profile than GLP-1 RA users). This gradient in study design, which runs from randomized trials and active-comparator cohorts through broad electronic health record analyses to spontaneous-reporting data, best explains why studies with the strongest control of baseline differences tend to be the most reassuring.

After the search closing date, a large Danish nationwide cohort and self-controlled case series study by Stanislaus and colleagues likewise reported no increased risk of suicide or suicide attempt with GLP-1 RA exposure, providing additional contextual support for the principal conclusion.^37^

### Generalizability

Randomized controlled trials almost universally excluded individuals with recent suicidality, severe depressive disorder, or unstable psychiatric illness, as serious mental illness is a standard exclusion criterion across the large phase 2/3 obesity and T2DM trial programs.^20^,^21^ The current literature therefore supports safety primarily in broader routine diabetes and obesity populations, not necessarily in patients with high baseline psychiatric vulnerability. That limitation is clinically important because obesity, diabetes, and many psychiatric disorders co-occur, and because GLP-1 RAs are increasingly being studied in severe mental illness, eating disorders, reward-related disorders, and substance use disorders. Dedicated evidence in psychiatric populations remains limited but is accumulating: a comprehensive analysis by De Giorgi and colleagues of 278 preclinical/mechanistic and 96 clinical studies did not identify a consistent signal of psychiatric worsening across mood, anxiety, psychotic, substance-use, and eating-disorder populations exposed to GLP-1 RAs, and highlighted several small studies suggesting possible benefit, while cautioning that most of this clinical evidence comes from small, short-duration, non-blinded studies that cannot exclude modest harm.^7^ A pilot randomized, double-blind, placebo-controlled trial of liraglutide 3.0 mg in people with schizophrenia, schizoaffective disorder, or first-episode psychosis likewise did not report an excess of psychiatric adverse events, although the trial was small and not powered to detect rare neuropsychiatric harms.^38^

### Tirzepatide

Evidence specific to tirzepatide remains limited but does not indicate increased neuropsychiatric risk. Real-world data suggest a lower risk of suicidal ideation or attempts compared with other anti-obesity medications,^30^ and clinical trial analyses from the SURMOUNT program demonstrate no increase in depression or suicidality.^21^ Follow-up durations remain relatively short, and existing studies may be underpowered to detect rare events. Continued long-term evaluation is warranted.

### Mechanistic Considerations

Mechanistically, the literature remains compatible with either null or beneficial psychiatric effects rather than direct harm. Central GLP-1 signaling originates from preproglucagon neurons in the caudal hindbrain, with projections to hypothalamic, brainstem, and limbic regions, and influences appetite, reward, neuroinflammation, stress, and cognition.^1^,^6^ A schematic of these proposed pathways is presented in Figure 2. There is emerging interest in possible antidepressant, cognitive, and addiction-related benefits.^7^,^8^,^39^,^40^ Although mechanistic hypotheses remain insufficient to justify therapeutic psychiatric claims, they help explain why improved mood and quality of life, restrained eating, and reduced emotional eating recur across RCTs instead of a coherent signal of deterioration.^18^,^41^

**Figure 2:**
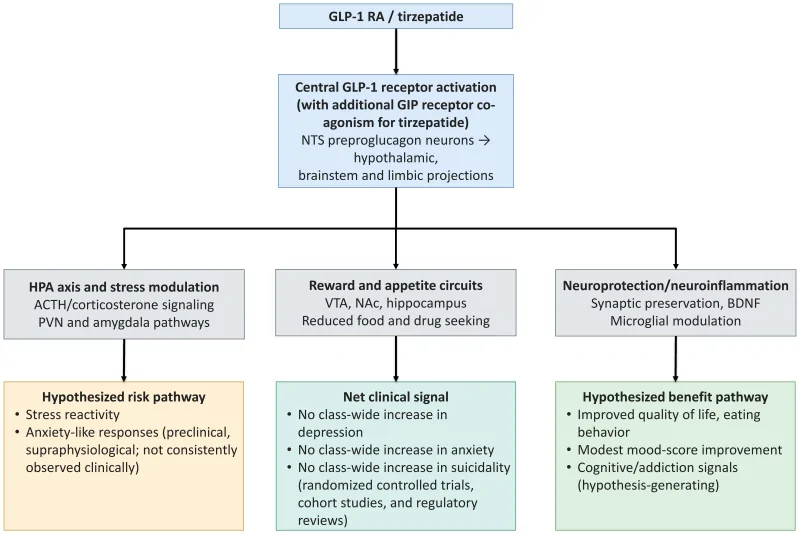
Proposed Mechanisms Linking GLP-1 Receptor Agonists to Neuropsychiatric Outcomes. Mechanistic pathways are hypothesized and integrate findings from preclinical and translational studies; they do not establish causality in humans. ACTH, adrenocorticotropic hormone; BDNF, brain-derived neurotrophic factor; GIP, glucose-dependent insulinotropic polypeptide; HPA, hypothalamic–pituitary–adrenal; NAc, nucleus accumbens; NTS, nucleus tractus solitarius; PVN, paraventricular nucleus; VTA, ventral tegmental area.

### Clinical Implications

Current evidence supports the use of GLP-1 RAs and tirzepatide without clear evidence of increased neuropsychiatric risk in the populations studied, although caution may be warranted in individuals with pre-existing psychiatric conditions or those receiving concomitant psychotropic medications, where closer monitoring may be appropriate.^34^,^38^ These findings are particularly relevant given the FDA’s January 2026 request to remove suicidal ideation/behavior label warnings,^5^ the EMA’s prior April 2024 conclusion that available data do not support a causal association,^4^ and the rapidly expanding use of these agents for both diabetes and obesity. These results may help inform clinical decision-making, support risk–benefit discussions with patients, and provide context for interpreting safety signals emerging from pharmacovigilance systems. The overall evidence and corresponding clinical judgments are summarized in Table 4.

**Table 4:** Summary of Evidence and Clinical Judgments.

Question	Evidence base	Certainty (GRADE)	Judgment
Do GLP-1 RAs increase suicide / self-harm in adults with diabetes or obesity?	RCT meta-analyses; active-comparator cohorts; regulatory reviews	Moderate	No consistent increase demonstrated
Do GLP-1 RAs increase incident depression or anxiety?	RCT meta-analyses; active-comparator cohorts	Low	No class-wide increase; comparator-sensitive variation remains
Does available tirzepatide-specific evidence show excess psychiatric harm?	Post hoc pooled RCT analysis; one real-world cohort	Low	No signal of increased risk in available evidence
Are pharmacovigilance signals sufficient to infer causality?	EudraVigilance; FAERS; WHO VigiBase	Very low	No—signal detection only
Should current practice change because of suicidality concerns alone?	Totality of evidence; EMA / FDA reviews	Moderate	No class-wide contraindication; clinically monitor higher-risk patients

EMA, European Medicines Agency; EudraVigilance, European Union adverse event reporting database; FAERS, FDA Adverse Event Reporting System; FDA, US Food and Drug Administration; GLP-1 RAs, glucagon-like peptide-1 receptor agonists; GRADE, Grading of Recommendations Assessment, Development and Evaluation; RCT, randomized controlled trial; VigiBase, World Health Organization global pharmacovigilance database; WHO, World Health Organization.

### Limitations

This review has several limitations. The evidence base was heterogeneous with respect to study design, populations, and outcome definitions. Observational studies are subject to residual confounding, while pharmacovigilance data are limited by reporting bias and the absence of denominator data. Follow-up durations in clinical trials were often relatively short, limiting the ability to detect rare or long-term neuropsychiatric outcomes. Evidence specific to tirzepatide remains limited. A review protocol was drafted before screening began but was not prospectively registered in PROSPERO, which we acknowledge as a methodological limitation. Finally, language restriction to English may have introduced a small risk of selection bias. Further long-term observational studies in psychiatrically vulnerable populations are needed to better characterize rare or delayed adverse events.

## CONCLUSIONS

Current evidence from RCTs, active-comparator observational studies, pharmacovigilance analyses, and regulatory reviews does not support an increased risk of depression, anxiety, or suicidality with GLP-1 RAs or tirzepatide in adults with T2DM or obesity, particularly in those without substantial pre-existing psychiatric comorbidities. The most credible evidence comes from randomized syntheses, recent active-comparator cohort studies, and regulatory reviews, all of which are broadly reassuring. Pharmacovigilance signals remain valuable for surveillance but should be interpreted as hypothesis-generating rather than causal. Future research should focus on patients with substantial psychiatric comorbidity, obesity-only populations, tirzepatide-specific long-term outcomes, and late rare-event surveillance.

## Supplementary Information



## Data Availability

All data analyzed in this review are derived from previously published studies cited in the references. Search exports and screening logs are available from the corresponding author on reasonable request.

## Abbreviations

AE: adverse event
CI: confidence interval
CPRD: Clinical Practice Research Datalink
DPP-4i: dipeptidyl peptidase-4 inhibitor
EMA: European Medicines Agency
FAERS: FDA Adverse Event Reporting System
FDA: US Food and Drug Administration
GLP-1 RA(s): glucagon-like peptide-1 receptor agonist(s)
GRADE: Grading of Recommendations Assessment, Development and Evaluation
HR: hazard ratio
OR: odds ratio
PHQ-9: Patient Health Questionnaire-9
PRISMA: Preferred Reporting Items for Systematic Reviews and Meta-Analyses
RCT(s): randomized controlled trial(s)
ROR: reporting odds ratio
RR: risk ratio
SGLT2i: sodium-glucose co-transporter 2 inhibitor
T2DM: type 2 diabetes mellitus
VigiBase: World Health Organization global pharmacovigilance database
