# Supplementary material for: Neuropsychiatric Safety of GLP-1 Receptor Agonists and Tirzepatide in Adults with Type 2 Diabetes Mellitus or Obesity
Source: Rambam Maimonides Med J. 2026 Oct 26;17(4):e0024. doi: 10.5041/RMMJ.10585 (PMC13719276; doi:10.5041/RMMJ.10585)
Supplement: Supplementary file 1 [file rmmj-17-4-e0024-supplement.pdf]

This appendix has been provided by the authors for the benefit of readers

Supplement to Neuropsychiatric Safety of GLP-1 Receptor Agonists and Tirzepatide in Adults with Type 2 Diabetes Mellitus or Obesity

Jani HT, Levine H, Patel A, Trivedi R, Patel XD. Neuropsychiatric Safety of GLP-1 Receptor Agonists and Tirzepatide in Adults With Type 2 Diabetes Mellitus or Obesity. Rambam Maimonides Med J 2026;17 (4):e0024. Review. doi:10.5041/RMMJ.10585

SUPPLEMENT A. DATABASE SEARCH STRATEGY FOR NEUROPSYCHIATRIC SAFETY OF GLP-1 RECEPTOR AGONISTS AND TIRZEPATIDE

Searches were conducted in PubMed/MEDLINE, Embase, Web of Science, and Scopus from inception through March 15, 2026, and the search is reported with reference to PRISMA-S. Search strategies combined controlled vocabulary and free-text terms for the intervention (GLP-1 receptor agonists and tirzepatide) and the outcomes (neuropsychiatric adverse events, depression, anxiety, suicidality). Limits: English language, human subjects, adults ≥18 years. No date restrictions were applied. Reference lists of included studies and relevant systematic reviews were also screened; this process identified no additional eligible records.

Concept blocks

Concept	Search Terms
Intervention	("glucagon-like peptide-1"[MeSH] OR "GLP-1 receptor agonist*" OR "GLP1 receptor agonist*" OR exenatide OR liraglutide OR dulaglutide OR semaglutide OR albiglutide OR lixisenatide OR tirzepatide OR Ozempic OR Wegovy OR Mounjaro OR Zepbound OR Saxenda OR Trulicity OR Victoza)
Outcomes	("suicide"[MeSH] OR "suicidal ideation"[MeSH] OR "self-injurious behavior"[MeSH] OR "depression"[MeSH] OR "anxiety"[MeSH] OR "mood disorders"[MeSH] OR "mental disorders"[MeSH] OR suicid* OR "self-harm" OR "self injury" OR depress* OR anxiety OR psychiatr* OR "mood disorder*" OR "neuropsychiatric adverse event*" OR "eating disorder*" OR PHQ-9 OR "Patient Health Questionnaire" OR C-SSRS OR "Columbia Suicide Severity Rating Scale")
Population	(adult* OR "middle aged"[MeSH] OR "aged"[MeSH] OR "diabetes mellitus, type 2"[MeSH] OR "obesity"[MeSH] OR "overweight"[MeSH] OR "type 2 diabetes" OR T2DM OR T2D OR diabet* OR obes* OR overweight)
Limits	English[Language]; Humans[MeSH]; "adult"[MeSH:NoExp] OR "middle aged"[MeSH] OR "aged"[MeSH]

Database-specific search strings as run

Database	Search string as run
PubMed/MEDLINE	("glucagon-like peptide-1"[MeSH] OR "GLP-1 receptor agonist*" OR "GLP1 receptor agonist*" OR exenatide OR liraglutide OR lixisenatide OR dulaglutide OR semaglutide OR albiglutide OR tirzepatide OR Ozempic OR Wegovy OR Mounjaro OR Zepbound OR Saxenda OR Trulicity OR Victoza) AND ("suicide"[MeSH] OR "suicidal ideation"[MeSH] OR "self-injurious behavior"[MeSH] OR "depression"[MeSH] OR "anxiety"[MeSH] OR "mood disorders"[MeSH] OR "mental disorders"[MeSH] OR suicid* OR "self-harm" OR "self injury" OR depress* OR anxiety OR psychiatr* OR "mood disorder*" OR "neuropsychiatric adverse event*" OR "eating disorder*" OR "PHQ-9" OR "Patient Health Questionnaire" OR "C-SSRS" OR "Columbia Suicide Severity Rating Scale") AND ("diabetes mellitus, type 2"[MeSH] OR "obesity"[MeSH] OR "overweight"[MeSH] OR "type 2 diabetes" OR T2DM OR T2D OR diabet* OR obes* OR overweight OR adult*) AND (English[Language] AND Humans[MeSH] AND ("adult"[MeSH:NoExp] OR "middle aged"[MeSH] OR "aged"[MeSH]))
Embase (Elsevier, embase.com)	('glucagon like peptide 1 receptor agonist'/exp OR 'exenatide'/exp OR 'liraglutide'/exp OR 'lixisenatide'/exp OR 'dulaglutide'/exp OR 'semaglutide'/exp OR 'albiglutide'/exp OR 'tirzepatide'/exp OR ('GLP-1 receptor agonist*' OR 'GLP1 receptor agonist*' OR exenatide OR liraglutide OR lixisenatide OR dulaglutide OR semaglutide OR albiglutide OR tirzepatide OR Ozempic OR Wegovy OR Mounjaro OR Zepbound OR Saxenda OR Trulicity OR Victoza):ti,ab,kw) AND ('suicide'/exp OR 'suicidal ideation'/exp OR 'automutilation'/exp OR 'depression'/exp OR 'anxiety'/exp OR 'mood disorder'/exp OR 'mental disease'/exp OR (suicid* OR 'self-harm' OR 'self injury' OR depress* OR anxiety OR psychiatr* OR 'mood disorder*' OR 'neuropsychiatric adverse event*' OR 'eating disorder*' OR 'PHQ-9' OR 'Patient Health Questionnaire' OR 'C-SSRS' OR 'Columbia Suicide Severity Rating Scale'):ti,ab,kw) AND ('non insulin dependent diabetes mellitus'/exp OR 'obesity'/exp OR 'adult'/exp OR ('type 2 diabetes' OR T2DM OR T2D OR diabet* OR obes* OR overweight OR adult*):ti,ab,kw) AND [english]/lim AND [humans]/lim AND [adult]/lim
Web of Science Core Collection (Clarivate)	TS=("GLP-1 receptor agonist*" OR "GLP1 receptor agonist*" OR exenatide OR liraglutide OR lixisenatide OR dulaglutide OR semaglutide OR albiglutide OR tirzepatide OR Ozempic OR Wegovy OR Mounjaro OR Zepbound OR Saxenda OR Trulicity OR Victoza) AND TS=(suicid* OR "self-harm" OR "self injury" OR depress* OR anxiety OR psychiatr* OR "mood disorder*" OR "neuropsychiatric adverse event*" OR "eating disorder*" OR "PHQ-9" OR "Patient Health Questionnaire" OR "C-SSRS" OR "Columbia Suicide Severity Rating Scale") AND TS=("type 2 diabetes" OR T2DM OR T2D OR diabet* OR obes* OR overweight OR adult*) Refined by: Languages = ENGLISH. Timespan: all years to 15 March 2026.
Scopus (Elsevier)	TITLE-ABS-KEY("GLP-1 receptor agonist*" OR "GLP1 receptor agonist*" OR exenatide OR liraglutide OR lixisenatide OR dulaglutide OR semaglutide OR albiglutide OR tirzepatide OR Ozempic OR Wegovy OR Mounjaro OR Zepbound OR Saxenda OR Trulicity OR Victoza) AND TITLE-ABS-KEY(suicid* OR "self-harm" OR "self injury" OR depress* OR anxiety OR psychiatr* OR "mood disorder*" OR "neuropsychiatric adverse event*" OR "eating disorder*" OR "PHQ-9" OR "Patient Health Questionnaire" OR "C-SSRS" OR "Columbia Suicide Severity Rating Scale") AND TITLE-ABS-KEY("type 2 diabetes" OR T2DM OR T2D OR diabet* OR obes* OR overweight OR adult*) AND (LIMIT-TO(LANGUAGE, "English"))

Abbreviations: C-SSRS, Columbia Suicide Severity Rating Scale; EMA, European Medicines Agency; FDA, US Food and Drug Administration; GLP-1, glucagon-like peptide-1; MeSH, Medical Subject Headings; PHQ-9, Patient Health Questionnaire-9; PRISMA-S, Preferred Reporting Items for Systematic Reviews and Meta-Analyses literature search extension; T2D and T2DM, type 2 diabetes mellitus.

Databases searched and records retrieved

Database	Records Identified	Search Date	Search Interface
PubMed/MEDLINE	398	15 March 2026	https://pubmed.ncbi.nlm.nih.gov/
Embase	342	15 March 2026	Elsevier Embase.com
Web of Science	261	15 March 2026	Web of Science Core Collection (Clarivate)
Scopus	223	15 March 2026	Elsevier Scopus
Total	1,224	—	—

Note: After de-duplication of 988 records, 236 records remained for title and abstract screening. Records were managed in Rayyan (Qatar Computing Research Institute) for screening and selection. Reference lists of included studies and recent systematic reviews were screened for additional eligible records; none were identified. Lixisenatide was searched as a member of the GLP-1 receptor agonist class and is included among the eligible agents in the manuscript; no lixisenatide-specific study met the inclusion criteria. The two regulatory reviews were identified from the EMA and FDA websites rather than from the bibliographic databases and are shown as a separate pathway in Figure 1.

SUPPLEMENT B. PER-STUDY RISK OF BIAS AND QUALITY APPRAISAL FOR NEUROPSYCHIATRIC SAFETY OF GLP-1 RECEPTOR AGONISTS AND TIRZEPATIDE (20 INCLUDED STUDIES + 2 REGULATORY REVIEWS)

RCTs and post hoc trial analyses were appraised with the Cochrane Risk of Bias 2 (RoB 2) tool. Comparative observational studies, including target-trial emulations and case-time-control designs, were appraised with Risk of Bias in Non-randomised Studies of Interventions (ROBINS-I). Pharmacovigilance and disproportionality analyses were qualitatively appraised because of inherent limitations of spontaneous reporting systems. Domain abbreviations: D1–D5 (RoB 2): D1, randomization process; D2, deviations from intended interventions; D3, missing outcome data; D4, measurement of the outcome; D5, selection of the reported result. Domains 1–7 (ROBINS-I): 1, confounding; 2, selection of participants; 3, classification of interventions; 4, deviations from intended interventions; 5, missing data; 6, measurement of outcomes; 7, selection of the reported result.

Rating scales: In both tools a rating of Low is the most favorable judgment (least risk of bias), and progressively more severe labels indicate greater risk of bias. RoB 2 uses Low risk of bias, Some concerns, and High risk of bias. ROBINS-I uses Low, Moderate, Serious, and Critical risk of bias, where Low denotes a study comparable to a well-conducted randomized trial and Critical denotes a study judged too problematic to provide useful evidence on the effect of the intervention. NI denotes no information. The scales are defined in full in the legends beneath Sections 1 and 3.

Section 1. RCTs and Post Hoc Pooled Analyses (RoB 2)

Study[Ref]	Design	Domains					Overall RoB	Key Concerns
		D1	D2	D3	D4	D5		
O'Neil 2017[16]	Pooled phase 2/3a RCTs of liraglutide 3.0 mg	Low	Low	Low	Some concerns	Some concerns	Some concerns	Post hoc harms pool; spontaneous SI capture less rigorous than later trials with C-SSRS/PHQ-9
Wadden 2024, STEP[20]	Post hoc pooled analysis of STEP 1, 2, 3, 5	Low	Low	Low	Low	Some concerns	Some concerns	Excluded participants with significant psychiatric history; rare event ascertainment
Wadden 2026, SURMOUNT[21]	Post hoc pooled analysis of SURMOUNT 1, 2, 3	Low	Low	Low	Low	Some concerns	Some concerns	Excluded participants with major baseline psychopathology; underpowered for rare events

Note: RoB 2 overall risk-of-bias ratings, from the most to the least favorable judgment: Low risk of bias, the trial is judged to be at low risk of bias for all five domains; Some concerns, the trial raises some concerns in at least one domain but is not at high risk of bias for any domain; High risk of bias, the trial is at high risk of bias in at least one domain, or has some concerns for several domains in a way that substantially lowers confidence in the result. The overall judgment takes the least favorable domain rating. D1–D5 are the five RoB 2 domains listed above; all abbreviations are defined at the end of this supplement.

Section 2. Trial-Based Meta-Analyses (synthesis-level appraisal; not re-appraised with RoB 2)

Study[Ref]	Design	Basis of Appraisal	Overall Certainty (GRADE)	Key concerns
Silverii (2024)[17]	Meta-analysis of 31 RCTs (84,713 participants)	Risk of bias of constituent trials as judged within the source synthesis	Moderate	Heterogeneous harms definitions; trials not designed around psychiatric outcomes
Pierret (2025)[18]	Meta-analysis of 80 RCTs (107,860 participants)	Risk of bias of constituent trials as judged within the source synthesis	Moderate	Variable outcome definitions across included trials
Ebrahimi (2025)[19]	Meta-analysis of 27 placebo-controlled RCTs (59,396 participants)	Risk of bias of constituent trials as judged within the source synthesis	Moderate	Rare events; imprecision; heterogeneous suicide/self-harm capture methods

Section 3. Observational Cohort and Case-Time-Control Studies (ROBINS-I)

Study[Ref]	Design	Domains							Overall ROBINS-I	Key Concerns
		1	2	3	4	5	6	7		
Tang (2024)[22]	US Medicare target-trial emulation	Mod	Low	Low	Low	Low	Mod	Low	Moderate	Residual confounding; claims-based outcome ascertainment
Ueda (2024)[23]	Scandinavian active-comparator new-user cohort	Mod	Low	Low	Low	Low	Mod	Low	Moderate	Confounding by indication; rare events
Shapiro (2025)[24]	UK CPRD active-comparator cohort	Mod	Low	Low	Low	Low	Mod	Low	Moderate	Residual confounding; outcome misclassification
Tang (2025)[25]	US Medicare target-trial emulation (depression)	Mod	Low	Low	Low	Low	Mod	Low	Moderate	Comparator-sensitive estimates; claims-based outcome
De Giorgi (2024)[26]	Propensity-matched cohort (semaglutide)	Mod	Low	Low	Low	Low	Mod	Low	Moderate	TriNetX EHR data; ICD coding ascertainment
Wang (2024)[27]	TriNetX cohort (obesity + T2DM replication)	Ser	Mod	Low	Low	Low	Mod	Low	Serious	Non-active comparator vulnerable to confounding by indication

Continued on next page.

Study[Ref]	Design	Domains							Overall ROBINS-I	Key Concerns
		1	2	3	4	5	6	7		
Gamble (2018)[28]	UK CPRD cohort	Mod	Low	Low	Low	Low	Mod	Low	Moderate	Earlier-era data; outcome misclassification
Bezin (2025)[29]	French SNDS case-time-control	Low	Low	Low	Low	Low	Mod	Low	Moderate	Self-controlled design; answers different question than parallel-group studies
Yu (2025)[30]	TriNetX tirzepatide real-world cohort	Ser	Mod	Low	Low	Low	Mod	Low	Serious	Non-active comparator; short follow-up
Kornelius (2024)[31]	Passive-comparator cohort (obesity)	Crit	Ser	Low	Low	Mod	Mod	Low	Critical	Outlier study; passive-comparator structure; severe confounding by indication

Note: ROBINS-I overall risk-of-bias ratings, from the most to the least favorable judgment: Low, the study is comparable to a well-conducted randomized trial; Moderate, the study is sound for a non-randomized study but not comparable to a well-conducted randomized trial; Serious, the study has one or more important problems; Critical, the study is too problematic to provide useful evidence on the effect of the intervention. The overall judgment takes the most severe domain rating, so a single Critical domain gives a Critical overall rating. Mod, Moderate; Ser, Serious; Crit, Critical. Domains 1–7 are the seven ROBINS-I domains listed above; all other abbreviations are defined at the end of this supplement.

Section 4. Pharmacovigilance and Disproportionality Analyses (qualitative narrative appraisal)

Study[Ref]	Database	Design	Overall Certainty	Key Limitations
Tobaiqy (2024)[32]	EudraVigilance	Descriptive case-series of psychiatric AE reports	Very low for causality	Descriptive only; no ROR/PRR; no denominator data; reporting bias
Chen (2024)[33]	FAERS	Disproportionality analysis (ROR/PRR)	Very low for causality	No denominator; notoriety bias; cannot establish incidence or causality
Schoretsanitis (2024)[34]	WHO VigiBase	Disproportionality analysis with subgroup interactions	Very low for causality	Subgroup analysis with co-prescribed psychotropics may reflect confounding by indication
Lu (2025)[35]	FAERS	Disproportionality analysis for neuropsychiatric AEs	Very low for causality	No denominator; weight-loss vs T2DM populations differentially captured

Section 5. Regulatory Reviews (decision relevance)

Source[Ref]	Date	Scope	Decision Relevance	Notes
EMA PRAC[4]	April 2024	Class-wide GLP-1 RA review	High	Concluded available evidence does not support a causal association
FDA Drug Safety Communication[5]	13 January 2026	91 placebo-controlled trials (107,910 participants) + Sentinel cohort (2,243,138 patients)	High	Requested removal of suicidal ideation/behavior label warnings

Note: Mod = Moderate; Ser = Serious; Crit = Critical; NI = no information. Under ROBINS-I the overall judgment takes the most severe domain rating; Kornelius 2024[31] is therefore rated Critical overall on the basis of critical risk of bias due to confounding. A rating of Critical means that the study was judged too problematic to provide useful evidence on the effect of the intervention. The three trial-based meta-analyses (Silverii 2024[17]; Pierret 2025[18]; Ebrahimi 2025[19]) were not re-appraised with RoB 2, because they are syntheses of individual trials rather than primary randomized controlled trials; for these, risk of bias as judged within each source synthesis was considered alongside the GRADE certainty rating, as described in the Methods. Each study was assessed using publicly available study reports.

Abbreviations: AE(s), adverse event(s); C-SSRS, Columbia Suicide Severity Rating Scale; CPRD, Clinical Practice Research Datalink; EHR, electronic health record; EMA, European Medicines Agency; EudraVigilance, European Union adverse event reporting database; FAERS, FDA Adverse Event Reporting System; FDA, US Food and Drug Administration; GLP-1 RA, glucagon-like peptide-1 receptor agonist; GRADE, Grading of Recommendations Assessment, Development, and Evaluation; ICD, International Classification of Diseases; PHQ-9, Patient Health Questionnaire-9; PRAC, Pharmacovigilance Risk Assessment Committee; PRR, proportional reporting ratio; RCT(s), randomized controlled trial(s); RoB 2, Cochrane Risk of Bias 2; ROBINS-I, Risk of Bias in Non-randomised Studies of Interventions; ROR, reporting odds ratio; SI, suicidal ideation; SNDS, Système National des Données de Santé (French national health data system); STEP, Semaglutide Treatment Effect in People with obesity; SURMOUNT, tirzepatide obesity trial program; T2DM, type 2 diabetes mellitus; TriNetX, federated electronic health record research network; UK, United Kingdom; VigiBase, World Health Organization global pharmacovigilance database; WHO, World Health Organization.
